# Perceptions and Rumors About the Ebola Virus Disease Vaccine in the Ituri and Kivu Provinces of the Democratic Republic of Congo

**DOI:** 10.29245/2578-3009/2023/S3.1111

**Published:** 2023-05-12

**Authors:** Ijeoma Igwe, Nkechi Onyeneho, Joseph C Okeibunor, Michel N’da Konan Yao, Tieman Diarra, Mamoudou Harouna Djingarey, Ibrahima Socé FALL, Abdou Salam Gueye

**Affiliations:** 1 University of Nigeria Nsukka; 2 World Health Organization; 3 Independent Consultant, Mali; 4 Independent Scientist, Niger

**Keywords:** Ebola virus disease, Vaccine, Rumors, Perceptions, Democratic Republic of Congo

## Abstract

Perceptions and rumors about vaccinations can contribute to vaccine hesitancy. This study aimed to examine perceptions and rumors about the Ebola vaccine during the 10th Ebola Virus Disease outbreak in the Ituri and North Kivu provinces of the Democratic Republic of Congo. Eight hundred randomly selected respondents were surveyed with a uniform structured questionnaire. Further, we collected qualitative data through focus group discussions and using in-depth interview guides. Results revealed several misperceptions and rumors about the vaccine, which led to some level of vaccine hesitancy and refusal among the people. The acceptance rate of the vaccine was 67.3% (below the 80% threshold needed to create herd immunity in the population). More of the urban population (31.3%) than the rural population (10.4%) accepted the vaccine. Refusals were largely due to fear that the vaccine could activate other diseases in the body and could even kill. Some feared that it was a conspiracy of the government to reduce the population in the study area through forced fertility control and death, among other such concerns. In conclusion, these rumors increased mistrust, which challenged the efforts of the government and its partners to safeguard the health of the people.

## Introduction

Immunization with effective vaccines against vaccine preventable diseases (VPDs) is of immense benefit for children, families, and communities even during complex disease outbreaks, such as the 2018 Ebola Virus Disease (EVD) outbreak in the Democratic Republic of Congo (DRC); it has been declared the best public health intervention^1^. It contributes to saving lives. Remarkably, it was the fundamental strategy for the eradication of smallpox. Polio is now on the brink of total eradication, with the African region recently certified free of all forms of the wild polio virus^2^. More children than ever before currently live healthy lives free of VPDs, thanks to immunization. Beyond the public health benefits, immunization has other social and development benefits. In terms of value for money, it has been shown that every dollar invested in vaccines during the last decade (2011−2020) resulted in an estimated return of 16 times the costs, considering treatment costs and productivity losses^1^. When considering broader economic and social benefits, the return on investment for immunization was 44 times the vaccination costs^3^. However, the effectiveness of vaccination as a public health intervention is affected by perceptions and rumors about vaccines in communities, despite efforts made to ensure the availability of effective vaccines to address even epidemic-prone diseases.

The EVD outbreak in West Africa witnessed a ramping up of vaccine development^4^, including animal studies and clinical trials^5–7^. The rVSV-ZEBOV was deployed for the first time during the April–July 2018 Ebola outbreak in the DRC^4^. In April 2018, Ebola emerged in a rural area of the Équateur province in the DRC and then spread to a populous urban center^8^. Relative to the West African outbreak, this recent DRC epidemic was swiftly contained after a total of 54 cases and 33 deaths^8^.

The vaccine was deployed again during a more complex and elongated EVD outbreak in the Ituri and Kivu provinces. This outbreak occurred in very difficult terrains, with complex security and political challenges. There are also pockets of community reluctance and the conflict setting continues to obstruct activities in some affected areas. The community’s resistance to the response team also complicated the response efforts, thus failing to achieve desired targets. There were attacks on the Médecins Sans Frontières (MSF)-run Ebola treatment centers on February 24 in Katwa and on February 27 in Butembo in the DRC. There was yet another attack on the Ebola treatment center in Biena on March 14, 2019. There have also been series of attacks on burial teams, infection prevention and control teams, as well as vaccination teams.

These attacks had significant immediate impact on lives and the risk of spreading the disease further. The exact reasons for the resistance and attacks on treatment centers as well as response teams present new lessons that the experiences from the West Africa outbreak may not have captured. In a sensitization meeting with religious leaders in Goma, the participants insisted that the Ebola scare is not real and alleged that it is a creation of the political class to subdue the people. Despite rumors about the nonexistence of Ebola and the attacks on the response teams, some of the people were vaccinated against Ebola. This depicts a mixed scenario of the acceptance of the Ebola prevention efforts. When driving past in WHO or partner vehicles involved in Ebola control, community members can often be seen praying for Ebola to leave their communities. This paper documents the perception and rumors about EVD vaccines during the 10^th^ EVD outbreak in the DRC.

## Study Design and Methods

### Study design

This study was designed to explore and document experiences and lessons around the response to the 10^th^ EVD outbreak in the North Kivu and Ituri provinces of the DRC. It adopted a cross-sectional design with mix-method techniques of data collection. The design allowed multiple windows of data harvesting, while the mixed-method approach brought the benefits of both quantitative and qualitative methodologies and guaranteed the integrity and robust interpretation and conclusions of this study.

#### Selection of the study area and population

The study was carried out in the North Kivu and Ituri provinces, where the 10^th^ EVD outbreak occurred in the DRC.

**Ituri** is one of the 26 provinces of the DRC. Its capital is the city of Bunia. The Ituri Rainforest is in this area. It is located northeast of the Ituri River and on the western side of Lake Albert. Ituri is a region of high plateau (2,000–5,000 m) that has a large tropical forest but also a savannah landscape. The district has rare fauna, including the okapi, the national animal of the Congo. As for flora, an important species is Mangongo, the leaves of which are used by the Mbuti to build their homes. Ituri is inhabited by different populations including the Alur, Hema, Lendu, Ngiti, Bira, and Ndo-Okebo, and one group constitutes the largest percentage of the total population. The Mbuti, a pygmy ethnic group, reside primarily in the Ituri forest near the Okapi Wildlife Reserve, although some Mbuti have been forced into urban areas by deforestation, over-hunting, and violence. The Kilo–Moto gold mines are partly located in Ituri. In the beginning of the 21st century, petroleum reserves were found by Heritage Oil and Tullow Oil on the shores of Lake Albert.

**North Kivu** (French: *Nord-Kivu*) is a province bordering Lake Kivu in the eastern DRC. Its capital is Goma. North Kivu borders the provinces of Ituri to the north, Tshopo to the northwest, Maniema to the southwest, and South Kivu to the south. To the east, it borders the countries of Uganda and Rwanda. The province has three cities: Goma, Butembo, and Beni, and six territories: Beni, Lubero, Masisi, Rutshuru, Nyiragongo, and Walikale. The province is home to the Virunga National Park, a World Heritage Site native to the endangered mountain gorillas. Except for the heightened insecurity and isolation due to rebel activities, North Kivu shares similar demographics with Ituri. The province is politically unstable and has been one of the flashpoints of the military conflicts in the region since 1998.

The **2018 or 10**^**th**^ **Kivu Ebola outbreak** began on August 1, 2018, when it was confirmed that four people had tested positive for the Ebola virus in the eastern region of Kivu in the DRC^9, 10^. The Kivu outbreak included Ituri Province, after the first case was confirmed on August 13. This outbreak started just days after the end of the 2018 Équateur province Ebola virus outbreak in the DRC^11^.

The affected province and general area are currently under a military conflict, which is hindering treatment and prevention efforts. WHO’s deputy director-general for emergency preparedness and response has described the combination of military conflict and civilian distress a potential “perfect storm” that could lead to a rapid worsening of the outbreak^12^. Owing to the deteriorating situation in North Kivu and the surrounding areas, the WHO, on September 27, raised the risk assessment at the national and regional levels from “high” to “very high”^12^.

The study population comprised adults aged ≥ 18 years living in the community, as well as response team members. A 2010 estimate put the population of North Kivu at 5,767,945. With an annual growth rate of 3.2%, the population in North Kivu in 2019 was reported to be 7,658,406 and 5,360,884 for the general and age ≥ 18 years populations, respectively. Meanwhile, a 2005 estimate put the population of Ituri at 4,037,561. An estimate of the population aged ≥ 18 years at approximately 74% resulted in 2,968,865 persons. For 2019, the populations were estimated as 6,275,305 and 4,392,714 for the general and age ≥18 years populations, respectively.

The response team consisted of over 10,000 persons. They belonged to different response pillars, namely, surveillance, risk communication, social anthropology, and vaccination. Others included infection prevention and control; treatment and care; safe and dignified burial; as well as security, logistics, and administration, among others.

#### Sample size estimation and sampling strategy

##### Sample size

With an assumed 50% chance of having accepted Ebola control interventions at a confidence interval of 95% and an error margin of 5%, a sample size of 384 was computed for the quantitative study. For the two provinces, the sample size was calculated at 768; this number was rounded up to 800 to allow for losses. The size of the qualitative study depended on the saturation of information after two sets of data were collected from each category of respondents.

##### Sampling strategy

A multi-stage sampling technique was adopted in selecting the communities, households, and respondents for this study. Two administrative areas (that are epicenters of EVD outbreaks within each province) were purposely selected. Ten communities were randomly chosen from each of the two administrative areas in the province.

##### Selection of households and respondents

The center of the selected community was the reference point where the team spun a pencil to determine the first route and first household, and thereafter moved to the right to identify the next household. This continued until the required number of households to be sampled was reached. Where there was a *cul-de-sac*, the step was retraced, and a turn to the left and then to the right was made to continue the sampling process.

Once an adult (≥ 18 years) was randomly selected in a chosen household for inclusion as a study participant, the sex of the participants was carefully alternated—that is, if in household #1 a male participant was selected, in the next household, the focus was on selecting a female participant.

### Methods

The study was conducted using a mix-method approach of qualitative and quantitative techniques. The methodology for data gathering involved in-depth interviews (IDIs), focus group discussions (FGDs), and a survey using structured questionnaires. This type of study requires a strong focus on individual actors rather than state actors.

#### Techniques of data collection

**FGDs** were conducted in North Kivu, as shown in Table 1. The same distribution was actualized for Ituri province.

A set of questions covering different thematic areas were developed to guide the discussions. The questions covered health care services in the community, awareness of and practices related to the EVD, as well as an assessment of the different pillars of the response interventions.

For the FGDs, 8−12 persons were selected for each session. A minimum of two FGDs were conducted in the selected communities. Separate FGD sessions were held for male and female participants in each of the communities. Overall, eight FGD sessions were conducted in each province.

**IDIs** were conducted in each community where FGDs were carried out. The IDIs were held with community/opinion leaders in the selected communities and the team leaders of the response pillars. Interviews were used to explore people’s opinions, views, and attitudes toward knowledge on practices and insights into the outbreak and response, as well as other socio-cultural factors that may influence people’s attitudes toward the response. The FGD guide was used for the IDIs, focusing on the thematic areas of interest to the evaluation.

**A structured questionnaire** was used to collect quantitative data from households. The questionnaire incorporated all the indicators that were used to answer the research questions and was structured based on the results of the qualitative study. It was categorized into the following sections: socio-demographic data, perception of health problems in the community, knowledge of the EVD, perceived epidemiology of Ebola in the communities, and sources of information on Ebola. The questionnaire also covered issues on communication and community engagement, infection prevention and control in the communities, vaccination, surveillance, and treatment and care. Other sets of questions covered the topics of safe and dignified burial, psychosocial issues, logistics, and security issues.

All interviews and discussions were tape-recorded, and detailed notes were taken simultaneously, including verbal citations. Tape-recorded interviews were transcribed. Observations were also recorded and, together with discussion and interviews, triangulated using the quantitative data to arrive at conclusions.

#### Training and pilot trials

All instruments were ***translated*** into Swahili and French, the common languages spoken in the communities, and back translated to English for clarity of meaning. In each province, ***10 research assistants*** with substantial experience in community interactive research, familiar with the use of qualitative and quantitative techniques, and knowledgeable about cultural sensibilities were recruited and trained for three days in Beni and another three days in Bunia on the study objectives and the use of the instrument for data collection. Training also included data entry into the Atlas.ti template (for qualitative data) and EPI INFO (for quantitative data). The instruments were reviewed after training, for clarity, understanding, and sensitivity. Each province had a ***supervisor*** who worked with the principal investigator on data quality monitoring, safety advisory, and ethical conduct of the research, including the management of informed consent procedures. The study was conducted first in Ituri, then in North Kivu. The lessons learned from Ituri were used to manage the process in the North Kivu area, a more challenging province in terms of security and logistics. The ***data analyst*** developed and pre-tested the template for data entry and analysis using the pilot test output. Given the short study period, data were collected using pencil and paper instead of android devices. The fieldwork took 20 days to complete for each province before analysis and report writing were conducted.

#### Data management

All **quantitative data** were double-checked by the researcher before computer input. Data were entered into EPI Info and processed using SPSS. Descriptive statistics were used to determine the proportions of various categories of respondents and indicators and for comparison. Frequency tables and graphic illustrations were used to present the data.

**Qualitative data** consisting of FGDs and IDIs were transcribed from audio records to text. All textual data were analyzed using the Atlas.ti software package. Data were analyzed according to themes corresponding to the indicators in the quantitative data and triangulated during presentation to enable complementary and analogous interpretation.

Given the continuous analytical process involved in qualitative analyses, it is important to note that the initial analysis of the key informant interviews and FGDs informed the final development of the structured questionnaire to be used in the study. This further enhanced triangulation between the two sets of data to be collected. The quantitative results contributed to our statistical conclusions, whereas the qualitative results placed emphasis on what was said and provided illustrative quotes that gave context and depth to the quantitative results.

#### Ethical considerations

The principle of do-no-harm was adhered to in the study. Informed study approval was obtained from the province, local administration, community, and household, while informed consent was obtained from all individuals involved in the study. The WHO/AFRO Ethics Review Committee provided ethical approval for this study. All researchers attended the mandatory training, which included substantial discussion on the relevant ethical issues. With a team constituting 50% female research assistants, same-sex interviews and FGDs were ensured. The assistants were also trained and mandated to comply with child protection and gender sensitivity guidelines in the process of data collection and visits.

## Results

The quantitative data were collected from a total of 800 respondents, with 50% constituting male participants. A participation rate of 50% was also ensured from each of the two provinces in the study, namely, Ituri and North Kivu. Meanwhile, the four health zones covered from the two provinces, namely, Beni, Bunia, Butembo, and Mandima, contributed 25% each to the data. A majority (70.0%) of the respondents were from urban areas and 68.1% were married (see Table 2).

**Figure 1:**
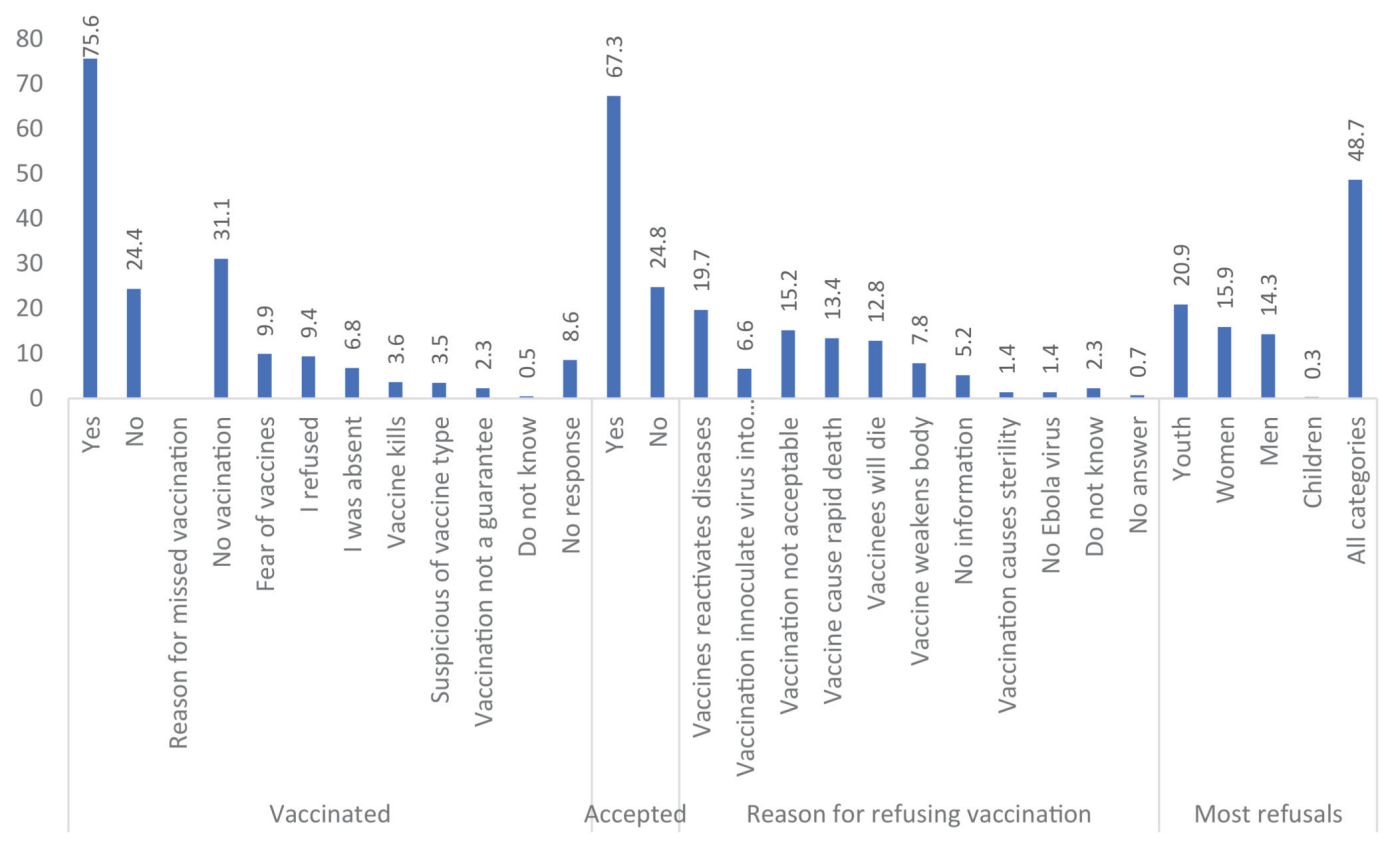
Ebola vaccine and vaccination acceptance during the EVD outbreak in the Ituri and Kivu provinces of the DRC

Two-thirds (67.3%) of the respondents accepted the vaccination as an effective form of intervention. However, less than a quarter of the people we met had been vaccinated; mainly the people we met who reside in urban areas were vaccinated, as shown in Figure 1. The most common reason mentioned for missed vaccination was no vaccination campaign (31.1%). Among those who refused vaccination were youth (20.9%), women (15.9%), and men (14.3%). The reasons for refusal include perceptions that vaccines reactivate diseases in the vaccinated (19.7%) and that vaccines cause death (13.4%).

**Figure 2:**
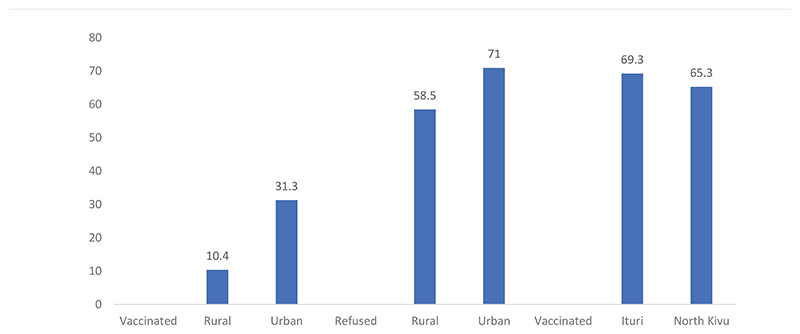
Vaccination with the Ebola vaccine during the EVD outbreak in the Ituri and North Kivu provinces of the DRC and the location of respondents (The Y axis refer to percentages)

More (31.3%) of the urban respondents than their rural counterparts were vaccinated. However, the results (see Figure 2) also revealed that more of the urban respondents (71.0%) refused vaccination. Fairly equal proportions of the respondents from the two provinces, Ituri (69.3%) and North Kivu (65.3%), were vaccinated.

Several other reasons were given in the qualitative arm of the study for refusal of the EVD vaccine, mostly due to misconceptions and rumors. Among these reasons, some people spoke of the lack of information about the vaccine. Others did not talk about the vaccine but blamed the existence of the EVD. Some spoke of the non-acceptance of the vaccine. In an interview with a community leader, he noted that, *some people say that, if we get vaccinated, we may have problems in the future. There are those who say that they have had the traditional vaccine and that there is no need to get another injection. They believe that the traditional vaccine is ash mixed with lemon and they say that this is an effective immunization when taken in a glass of water in the morning, at noon, and in the evening, for three days. Some charlatans say they have their traditional vaccine too. So, someone who believes in these people and is taking the immunization shots will not have the courage to get vaccinated*.

The “forces of evil” as a cause of the EVD emerged as an important perception in both regions. Mainly articulated in FGDs involving older participants, this perception was also highlighted by community health workers and traditional healers, who have a keen understanding of their communities.

Perceptions and experiences about the EVD vaccination were explored in several ways during the FGDs and interviews. Thematic areas included decision-making within households related to household protection against the EVD and vaccination with EVD vaccines and perceived benefits of and constraints to EVD vaccination.

Results indicated that decision-making related to vaccination in the households varies. In rural areas, parents said the decision usually fell to the father or to another adult male household member. In urban areas, parents generally agreed that mothers were the ones who usually decided whether to vaccinate while adult members of the household were free to accept or refuse vaccination. In the case of children, it is the mother who mostly decides, because when they visit the clinic, they are taught about various types of vaccinations and their sequencing, including dates.

Some study participants had more nuanced answers on the question of who might decide whether to have a household member vaccinated. They noted that in some instances, the family, including the extended family, may be involved in such a decision. In many situations, who makes the decision may depend on who is the main income earner, how busy the mother is at home, and who can provide transportation for the mother and child. Some participants noted that the family may also be influenced by a broader network of people that includes neighbors, community leaders, and health workers.

While parents were generally positive about childhood immunization programs in the DRC, they also provided insights into why people were suspicious and reluctant to accept the EVD vaccine. In this regard, many pointed to a lack of understanding among some people of the true existence of the EVD and the genuineness of the vaccine. Some people who are informed that there is a vaccine being given choose to not receive it: “*They say ‘what is it for?’ They do not understand what it is for, therefore do not let their children go for it*.” (FGD: Adult male participant, Beni).

A community health worker in Butembo offered another perspective, pointing to the role of the older populations.

*They may argue that they have been living with the disease for decades and have never been asked to be vaccinated and were not vaccinated and yet they have remained alive to their old ages and healthy*. (FGD: community health worker, Butembo)

Parents in FGDs in both Bunia and Mandema voiced a variety of concerns about the potential side effects of vaccination and about perceived injection practices. Side effects described as common included soreness at the vaccination site and slight fevers, while serious side effects included abscesses at the vaccination site, which were attributed to poor injection techniques which could even lead to death. The parents said that they preferred to be served by experienced service providers as opposed to “children in the research team,” and some participants said they were concerned that needles and syringes were reused on different children.

In a related concern, several participants suggested that people may worry about getting vaccinated because they fear the child might become infected with other diseases including HIV through unsafe injection practices. Some highlighted fears that the government may be using vaccines to kill the adult population in the area and sterilize young female children or to reduce the population. Study participants noted that some religious denominations also forbid vaccination.

Participants in several parent FGDs suggested that some women seemed to be the focus of negative reactions at vaccination centers for reasons that had to do with their parenting choices. There were reports of some mothers being scolded by the vaccination team. For their part, members of the vaccination team spoke of feelings of frustration when people refuse to come in for vaccination. Such frustration could lead to unfriendliness or scolding. Some members of the vaccination team and many parents described households where people are unvaccinated as “lazy,” “ignorant,” or “difficult.” A Beni health worker captured the frustrations of the vaccination team:

*They are careless people. Continued visitation or education through a close friend will help change them. Some of the people refusing do not have proper knowledge of the disease and the benefits of EVD vaccines. Education should be done through kindred meetings called by local community leaders and other administrators and churches for them, to help protect the life in the community*. (FGD: community health worker, Beni)

Rumors about the deadly effects of the vaccine and doubts about the true status of the disease also posed challenges and constraints on efforts to get the people vaccinated against the EVD. Some of these rumors are compiled and classified in Table 3.

Some of the information about prevention is used to support and substantiate rumors. For example, the presence of the virus in the semen of some men who recovered from the EVD and the precautionary use of condoms is considered by some as a policy for birth control.

## Discussion

Vaccination is an important part of the response to the Ebola epidemic, and immunization is not only linked to the availability of shots but also depends on acceptability by the community members (Chowell et al., 2019). Dr Moeti, the Regional Director of WHO in the African region stated “with effective vaccines at hand and the experience of DRC health workers in Ebola response, sufficient information on the vaccine has been supplied to the community by the response teams”^13^.

However, rumors are likely to influence the decision making of some community members on whether to get vaccinated. With support from WHO and other partners and donors, the country has become expert in mounting effective Ebola response, the UN agency noted^13^. It thus appears that information on the vaccine must consider everything that is said by the population. If a rumor is believed, there is nothing we can do about it. The abovementioned rumors come from all categories of people who were encountered throughout this research. They are community members, community leaders in various functions, religious people, members of associations, healthcare workers in various occupations, municipal workers, administrators, and other stakeholders. Furthermore, these rumors come from both men and women.

Rumors and conspiracy theories can lead to vaccine hesitancy and even vaccine refusals. Negative claims about vaccine effectiveness have affected vaccine uptake in other public health programs in the past. In Nigeria, for instance, the boycott of the polio vaccine due to rumors that the vaccine caused infertility led to increased polio cases in Nigeria, Pakistan, and Afghanistan^14, 15^. Rumors often challenge health interventions of governments and their partners such as the WHO^16^. An individual’s belief of a piece of information as misinformation or not is dependent on the individual’s level of health literacy and risk perceptions. However, continuous exposure to social media and online anti-vaccine movements may worsen the situation and influence people to share and communicate vaccine misinformation and conspiracy theories^17–19^. Lack of confidence in disease-specific vaccine candidates may lead some people to delay or refuse the new vaccine, potentially disrupting national and international control efforts^20^. Culturally compelling and context-appropriate information, supported by credible sources, are required to prevent vaccine misinformation^21^.

In a study, Islam et al.^22^ “identified 637 COVID-19 vaccine-related items: 91% were rumors and 9% were conspiracy theories from 52 countries. Of the 578 rumors, 36% were related to vaccine development, availability, and access, 20% related to morbidity and mortality, 8% to safety, efficacy, and acceptance, and the rest were other categories. Of the 637 items, 5% (30/) were true, 83% (528/637) were false, 10% (66/637) were misleading, and 2% (13/637) were exaggerated.” The key conclusion from the study was that rumors and conspiracy theories may cause mistrust, further leading to vaccine hesitancy.

In the current study of rumors about the EVD vaccine in the DRC, there were many rumors about the EVD vaccine and vaccination. Some of these had to do with the suspicion that the vaccine could kill and transmit diseases. Others had to do with human reproduction. It was believed that the vaccine kills reproductive cells in human. Several issues can be drawn from these rumors:

- These rumors are not about the behavior of the members of the vaccination team.
- The rumors represent a suspicion of the national authorities. They mention national authorities, similar to the rumors about the disease.
- Rumors create fear as they pertain to the future consequences of the vaccine.
- Changes in vaccination targets have not been understood and have been wrongfully interpreted through rumors.
- The notion of hidden motives is present, and it is perceived from different angles.
- The vaccine is not presented as a means to protect but rather to weaken. This is expressed in several rumors about the reduction of life expectancy and about the vulnerability of the body.
- Vaccines are perceived as their exact opposite. Instead of protection, they are seen as a source of danger. This danger would implicate the deterioration of the state of health by awakening diseases that were inactive. In some cases, this would happen with all diseases, not just the ones that are inactive. This danger could lead to the death of multiple people.
- The vaccine is presented as if it was meant to cause the death of an entire community or population. Thus, it would be intended to exterminate and not to protect.
- The vaccine is considered to have a political purpose. It is said that it is destined to prevent elections. It is also seen as a weapon used by the authorities to achieve undeclared objectives.
- The vaccine is seen to compromise the future in several ways. It would prevent reproduction or compromise the future of the next generations, as it would be the source of a reduction in the intellectual capacity of the children born to vaccinated people.
- The vaccine would make individuals less capable of working and reduce productivity. It would lead to weakening the individual or the body and is thus perceived as having an incapacitating effect. This incapacitation happens with the injection of the disease.
- There are doubts, suspicions, and questions about the vaccine. People say the vaccine is different from how it used to be. Thus, it is considered as not being the same for everyone. The real vaccine would then be for the *response* teams, and the fake vaccine for the population. Changes in targets are considered suspicious and are not seen as beneficial to results of coverage and protection. Doubts are expressed about the vaccine, also considering it to be fake. The same is true for efficacy. Therefore, a vaccinated person would not be more protected against the disease than one who is not vaccinated.
- The principle of protection is however not always questioned, similar to that of prevention by a vaccine. It is even said that a traditional vaccine to fight the EVD exists, and its posology is also supplied.

In conclusion, through the rumors, the question of trust arose, and introduced some level of vaccine hesitancy in the communities. However, it does apply to national authorities, who are more visible, and the intentions attributed to them.

## Figures and Tables

**Table 1:** Distribution of Participants in the In-Depth Interviews and Focus Group Discussions by Province

Target	North Kivu Province				Ituri Province			
	Butembo		Beni		Mbuti		Bunia	
	IDI	FGD	IDIdf	FGD	IDI	FGD	IDI	FGD
Pillar leads	All		All		All		All	
Pillar members	2/ pillar		2/ pillar		2/ pillar		2/ pillar	
Community leaders^1^	≥2/ community		≥2/ community		≥2/ community		≥2/ community	
Leader of survivor group	≥2/ community		≥2/ community		≥2/ community		≥2/ community	
Adult male participants		≥2 groups		≥2 groups		≥2 groups		≥2 groups
Adult female participants		≥2 groups		≥2 groups		≥2 groups		≥2 groups
Male youth		≥2 groups		≥2 groups		≥2 groups		≥2 groups
Female youth		≥2 groups		≥2 groups		≥2 groups		≥2 groups
Survivors		≥2 groups		≥2 groups		≥2 groups		≥2 groups

1 Here, the community leaders include traditional, political, religious, and opinion leaders in the community.

**Table 2:** Demographic Characteristics of Respondents

Characteristics	Category	Number	Percentage
Sex	Male	400	50.0
	Female	400	50.0
Province	Ituri	400	50.0
	North Kivu	400	50.0
Zone	Beni	200	25.0
	Bunia	200	25.0
	Butembo	200	25.0
	Mandima	200	25.0
Health area	Beni	160	20.0
	Bunia	100	12.5
	Butembo	100	12.5
	Kyondo	100	12.5
	Mandima	200	25.0
	Oicha	40	5.0
	Rwampara	100	12.5
Locality	Rural	240	30.0
	Urban	560	70.0
Marital status	Single	208	26.0
	Married	545	68.1
	Divorced	11	1,4
	Widowed	36	4.5

**Table 3:** Compilation of Rumors About the EVD Vaccine in the Ituri and North Kivu Provinces of the DRC

S/N	Areas of rumors	Descriptions
**1**	**Vaccine to influence demographics**	The vaccine reduces life expectancy. Vaccinated people will live no longer than five years. The introduction of the vaccine is a new strategy to exterminate the population following the failure of the Allied Democratic Forces (ADF) mission. The Rwandans want to kill the people of the East with the vaccine. Healthcare workers put products in the vaccine to spread the disease. Kabila wants to exterminate the people of the East with the vaccine. The vaccine weakens people, who will die in droves. People will die three years after vaccination. If you get vaccinated, the Nalu rebels do not kill you. They leave you and say you are already dead. ADF rebels met a group of people. They asked for those who have not been vaccinated. They said to the others: “You, who have been vaccinated, you are already dead.” After getting vaccinated, the person dies within a few years (two or three years). The vaccine was brought in by a politician to cause harm. The vaccine is meant to eliminate the entire Congolese population. The vaccine shortens life expectancy.
**2**	**Vaccine to reduce intelligence**	15. The vaccine has a negative impact on intelligence. 16. The vaccine creates psychosis and leads to mental health problems. 17. The vaccine reduces the intelligence of children. 18. The vaccine weakens the body. 19. The vaccine weakens the community. 20. The vaccine causes incapacity five years after its administration
**3**	**Skepticism about the vaccine**	21. The vaccine is fake. 22. The vaccine is an experiment, and it must be tried on animals first. 23. The vaccine is an experiment on the Nande population. 24. The vaccine was restricted to contacts, so why has it been liberalized? 25. The first vaccine had conditions for its administration. Why is this not the case for the second vaccine? There must be other reasons why.
**4**	**Reproductive vaccine**	26. The vaccine destroys reproductive cells (sperm). 27. The vaccine prevents childbearing. 28. The vaccine makes young girls and young boys infertile
**5**	**Existence of a good and a bad vaccine**	29. There are two kinds of vaccines: the best is given to the authorities and healthcare workers; the worst is given to the population. 30. The old vaccine was good, but the new one is not, it is blood. 31. The vaccine has been altered. The first one was good.
**6**	**The vaccine transmits the EVD**	32. The vaccine is a disease that is being distributed among the population. 33. The vaccine awakens hidden diseases in the body. 34. The vaccine is the disease that is injected. 35. The vaccine activates all the diseases.
**7**	**Other remarks about the vaccine**	36. I was not vaccinated, and I am alive. 37. The vaccine is not available to everyone, only to “contacts.” 38. One family refused the vaccine. Someone from the team went to the family's toilet. Afterward, a family member went to the toilet, and was infected with the disease. This happened in the Mabolio neighborhood. 39. There is an effective traditional vaccine made of ash mixed with lemon. You must take one glass of water in the morning, one at noon, and one in the evening. 40. Charlatans will say they have their vaccine.

## Data Availability

The data supporting the findings of this study are not publicly available because they contain information that could compromise the privacy of the research participants. The data are available from the corresponding author (Joseph Okeibunor), upon reasonable request.
